# Exceptional response to alpelisib in a heavily pretreated patient with metastatic breast cancer carrying a *PIK3CA* mutation outside SOLAR-1 eligible mutations: a case report

**DOI:** 10.2340/1651-226X.2026.46486

**Published:** 2026-09-23

**Authors:** Karolina F. Larsson, Annabelle Forsmark, Henrik Fagman, Maja Drakenberg Danese, Maria Kaire, Matilda Liljedahl, Christoffer Vannas

**Affiliations:** a Department of Oncology, Institute of Clinical Sciences, Sahlgrenska Academy, University of Gothenburg, Gothenburg, Sweden; b Department of Oncology, Sahlgrenska University Hospital, Gothenburg, Region Vastra Gotaland, Sweden; c Department of Laboratory Medicine, Institute of Biomedicine, University of Gothenburg, Gothenburg, Sweden; d Department of Clinical Pathology, Sahlgrenska University Hospital, Gothenburg, Region Vastra Gotaland, Sweden

**Keywords:** Breast neoplasms, neoplasm metastasis, precision medicine, molecular-targeted therapy, PIK3CA gene, phosphatidylinositol 3-kinases

## Introduction

Targeted therapies have improved outcomes in metastatic breast cancer (MBC) [1]. Alpelisib, a selective PI3K inhibitor, has recently been introduced as a treatment option [2]. However, it has not yet been widely used in Sweden [3], and its role in MBC remains to be established.

The median survival of around 3 years in MBC seen in the early 2000s has improved with therapeutic advances, especially for selected subtypes [4]. The concept of targeted therapies was raised by Paul Erlich already in the late 19th century, with research focusing on finding ‘magic bullets’ to target infectious diseases and cancer [5, 6]. Within breast cancer, the first targeted drug came into clinical use in the 1970s, with the approval of tamoxifen, providing exceptional benefit in both the adjuvant and the metastatic setting [7].

Oestrogen receptor (ER)-positive, Human Epidermal Growth Factor Receptor 2 (HER2) negative (ER+HER2-) breast cancer represents 68% of MBC cases in Sweden [8]. Since the introduction of Cyclin-Dependent Kinase 4/6 inhibitors (CDK4/6i) in combination with endocrine treatments, median survival for this subtype has improved to 5 years [9]. However, resistance remains a challenge, often mediated by somatic *PIK3CA* mutations in the PI3K pathway occurring in approximately 40% of patients [2, 10, 11]. Adding PI3Kα inhibitor alpelisib to fulvestrant as a second line treatment has shown an improved progression-free survival compared with fulvestrant alone (11.0 months vs. 5.7 months) in the SOLAR-1 study [2]. Nevertheless, alpelisib has not yet been widely adopted in clinical practice in Sweden [3]. This is partly due to difficulties in defining its role within an evolving treatment algorithm, since this study was conducted before the widespread use of CDK4/6 inhibitors [12]. Notably, prior exposure to CDK4/6i was limited in the SOLAR-1 trial, with only 5–6% of enrolled patients having received such treatment [2]. Evidence from the phase II BYLieve study subsequently demonstrated the activity of alpelisib in combination with fulvestrant in patients progressing on an aromatase inhibitor and a CDK4/6 inhibitor, with 54% of patients alive and progression-free at 6 months [13]. Despite its non-randomised design, this study provided important supportive evidence and contributed to the endorsement of this treatment approach [14].

Additionally, the timing, patient selection and the availability of molecular testing have been unclear as well as concerns about the benefit-to-toxicity ratio of alpelisib, as recently reported by us [3]. Furthermore, the patients were required to have at least one of 11 described *PIK3CA* mutations to be eligible [2]. The selection of these mutations lacks a clear biological rationale and seems to be based mainly on their prevalence.

In this case report, we describe the use of alpelisib in combination with fulvestrant in a heavily pretreated patient with ER+HER2-MBC, carrying a *PIK3CA* mutation outside the SOLAR-1 eligible mutations.

## Case presentation

**Figure 1:**
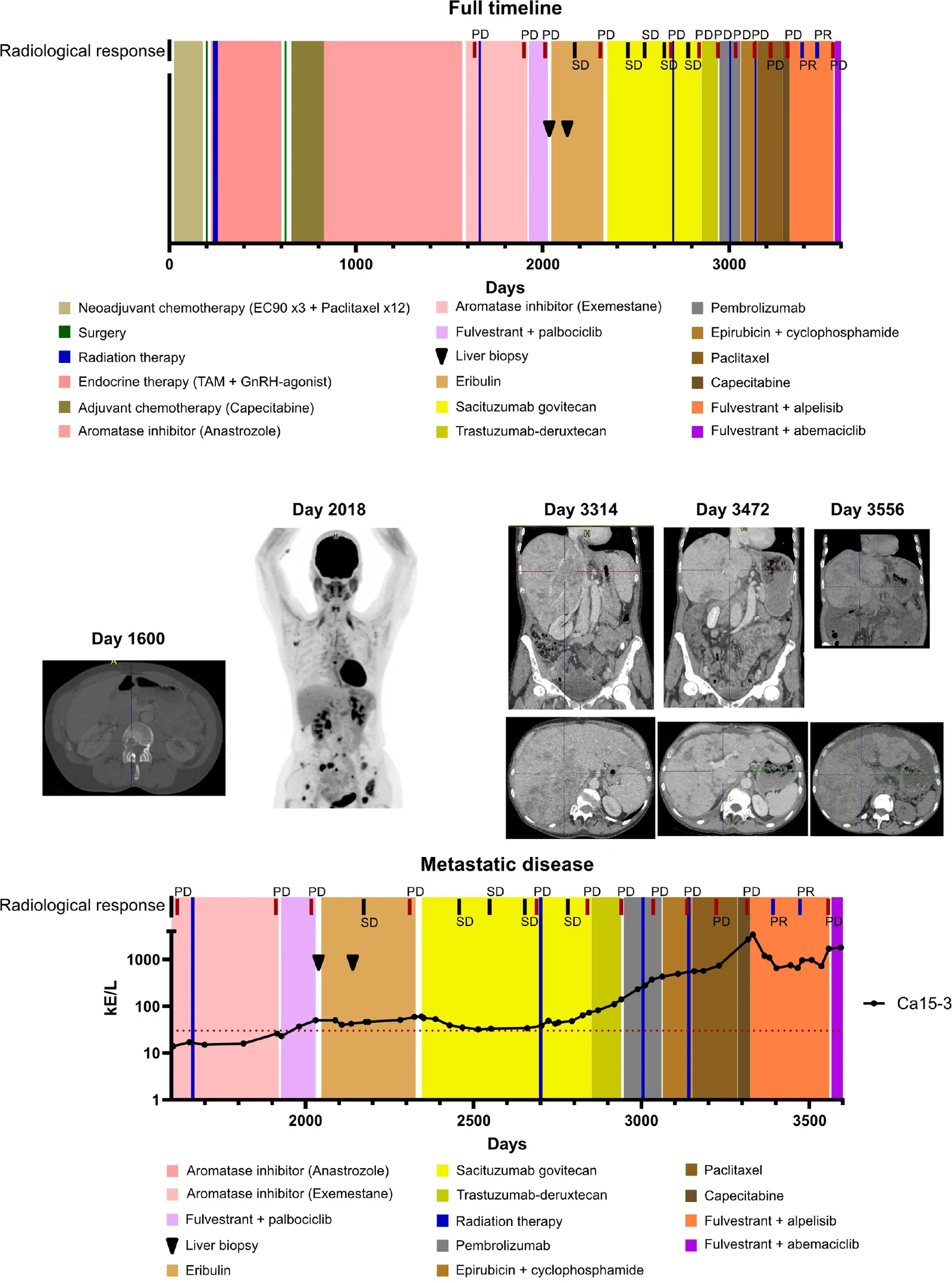
Treatment timeline. Results from radiological images (above) and Ca15-3 trend (below), describing the clinical exceptional response to treatment with alpelisib and fulvestrant in a heavily pretreated patient with metastatic breast cancer harbouring a *PIK3CA* mutation outside the SOLAR-1 panel. The red-dotted line indicates the lower limit of normal for Ca15-3. PR: partial response; SD: stable disease; PD: progressive disease.

In 2016, a female patient at the age of 43 was diagnosed with stage II ER+HER2- breast cancer (Figure 1, day 0). Neoadjuvant chemotherapy with anthracyclines and taxanes was given, followed by surgery, radiotherapy and tamoxifen in combination with a GnRH-agonist. In 2018, a regional recurrence (Figure 1, day 600) was treated with a second surgery, adjuvant capecitabine and the aromatase inhibitor anastrozole. In 2020, she developed a bone-only oligometastatic distant recurrence (Figure 1, day 1600). Treatment was switched to exemestane, and stereotactic radiotherapy was given. Liver and lymph node metastases were diagnosed in 2021 (Figure 1, day 2018). Beyond first line, the patient received the following treatmens in subsequent years: fulvestrant and palbociclib, eribulin, sacituzumab govitecan, trastuzumab deruxtecan, pembrolizumab within the Phase I study Keynote-D20 (NCT04752826) [15], epirubicin and cyclophosphamide, and paclitaxel and capecitabine (Figure 1). Most treatments had modest or no effect, except for sacituzumab govitecan, which produced a response with a stable disease for 16 months. From 2020 to 2025, the disease remained relatively stable without major symptom burden.

**Figure 2:**
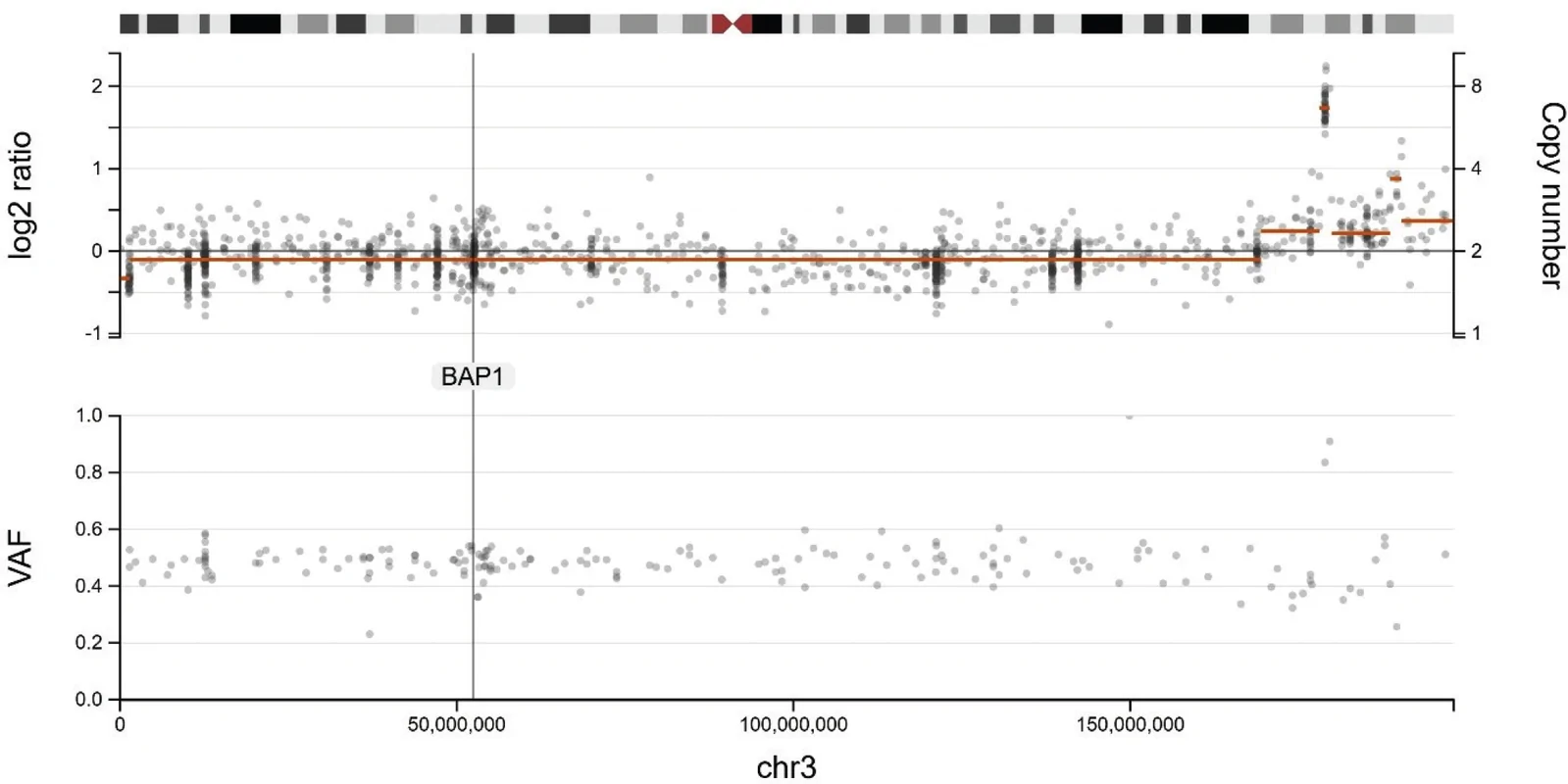
Comprehensive genomic profiling (Genomic Medicine Sweden 560 panel) showing amplification of the *PIK3CA* locus at 3q26.32. The black line at 0 represents the diploid genomic baseline, whilst the red line indicates the average copy number across chromosome 3.

In 2022, the patient was included in the explorative clinical MEGALiT trial (NCT04185831), aiming to investigate the feasibility and clinical benefit of genomics-based precision medicine in advanced cancers. Her case was subsequently included and described in the trial’s primary publication [16]. Analysis of a biopsy using FoundationOne®CDx [17] from a newly diagnosed liver metastasis revealed a likely oncogenic mutation in *PIK3CA* (K111del) as well, a low-level amplification of the gene (Figure 1, day 2138 and Figure 2). This did not meet trial basket criteria for study-specific treatment in the MEGALiT trial. Since the mutation was not one of the hotspot mutations defined in the SOLAR-1 panel [2], treatment with alpelisib was not initiated at this point. In retrospect, the same *PIK3CA* mutation had been identified already in 2021 (Figure 1, day 2033), in a previous liver metastasis, during routine clinical testing for alpelisib eligibility.

In 2025, the patient experienced rapidly increasing cancer-related symptoms with severe pain in the axial skeleton, nausea, cachexia and general oedema. Evaluation revealed massive progression of liver and bone metastases, a marked increase of tumour marker Ca15-3, elevated liver enzymes and treatment refractory hypercalcemia (Figure 1, day 3314). The impending liver failure was life-threatening at this stage, and all available standard treatments had been exhausted. Based on the identification of a *PIK3CA* mutation and the absence of standard treatment options at the time, off-label treatment with alpelisib plus fulvestrant was initiated following multidisciplinary discussion and individualised clinical assessment, despite prior progression on fulvestrant. The treatment was administered outside the MEGALiT trial protocol, with informed patient consent and approval from the Head of Department. Within days of treatment initiation, clinical response could be noted with decreased fatigue, decreased symptoms related to hepatomegaly, relief of nausea and feeding difficulties. After 6 weeks, tumour marker Ca15-3 and liver enzymes had dropped considerably, and serum calcium was normal. Radiological evaluation after 10 weeks showed a partial response in the liver and stable disease in bone and lymph nodes (Figure 1, day 3472). Alpelisib was well tolerated, apart from mild to moderate hyperglycaemia, an on-target side effect that was managed with metformin. Her overall well-being remained improved until a clinical and radiological progression was observed 8 months later (Figure 1, day 3556). The patient passed away shortly after the progression.

## Discussion

This case report illustrates a substantial clinical response to alpelisib in a patient carrying a *PIK3CA*-mutation outside the SOLAR-1 eligible mutations [2]. The response is exceptional, given that the treatment was initiated after heavy previous treatments, in a situation with life-threatening liver failure, a setting in which the best expected outcome would be disease stabilisation. Notably, across the nine prior lines of oncologic therapies, the best observed outcome was stable disease. This case adds to a small but growing body of evidence linking response to alpelisib beyond SOLAR 1- mutations [18, 19].

At present, alpelisib use often relies on predefined diagnostics (e.g. PIK3 Therascreen), none of which includes more than the SOLAR-1 11 hotspot mutations [19]. A review study exploring the coverage of the Therascreen gene list reported that it would capture only 72% of all breast cancer *PIK3CA* mutations [20], indicating a risk that potentially oncogenic mutations may be missed. In addition, the optimal timing of screening for mutations is of great importance. A recent Swedish study of endocrine-resistant recurrent breast cancer found that mutations were more common in relapsed than primary tumours [21], highlighting the need for repeated biopsies. The patient in our case had the *PIK3CA* mutation screened from two different liver biopsies in the clinically endocrine-resistant setting.

One of the non-hotspot mutations picked up in the Swedish study cohort, and (E110del) has been reported to increase signalling in the PI3K pathway in cell lines [22, 23] and is interestingly located next to the mutation of our case (K111del). In combination with the low-level amplification observed in our patient, these findings suggest a dependence on PI3K pathway signalling, which may explain the exceptional response.

Phosphoinositide 3-kinases (PI3Ks) are heterodimers consisting of a catalytic subunit (p110) and a regulatory subunit (p85) [24]. The *PIK3CA* gene, which encodes the p110α subunit, is one of the most frequently mutated oncogenes in cancer [25]. Mutations mainly occur in two hotspot regions: the helical domain and the kinase domain [19, 26]. The natural transition from an inactive to an activated form of p110α can be separated by four distinct events, and oncogenic mutations enhancing any of these events cause a constant activation of the enzyme [26]. Accordingly, *PIK3CA* hotspot mutations all affect at least one of these events. The *PIK3CA* mutation of our case (K111del) maps to the linker between the adaptor-Binding Domain and Ras-Binding Domain, a region in which mutations have been shown to enhance two of these activating events [26]. The *PIK3CA* K111 mutational site is infrequent but included amongst the top 25 most prevalent sites across different cancers [27]. In breast cancer specifically, a substitution at K111 is a rare event with a frequency below 1% [19, 20].

Although alpelisib was the only approved PI3K pathway-targeted therapy available at the time of treatment, the subsequent introduction of capivasertib, an oral pan-AKT inhibitor [28], has expanded the therapeutic landscape and further emphasised the importance of biomarker-driven treatment in endocrine-resistant HR-positive/HER2-negative MBC. Notably, capivasertib has demonstrated the greatest clinical benefit in patients whose tumours harbour alterations in the PI3K/AKT/PTEN pathway, including *PIK3CA* mutations [28].

In summary, this case illustrates how genomics-driven cancer treatment can provide substantial clinical benefit. Although the mutation had been identified earlier, the therapeutic potential of alpelisib was only recognised through a genomics-guided drug repurposing study [16, 29].

## Conclusion

We describe an exceptional response to alpelisib and fulvestrant in a heavily pretreated patient with ER+HER2- MBC harbouring a *PIK3CA* mutation outside the SOLAR-1 eligible mutations. This case report highlights the need for robust diagnostics capable of detecting *PIK3CA* mutations as well as the necessity to further study the use of *PIK3CA*-inhibitors in MBC.

## Patient perspectives

The patient in this case is included as a full co-author (AF). She was a senior scientist with a PhD in Cell and Molecular Biology and contributed substantially to manuscript preparation. She expressed a wish for her case to be published, reflecting her commitment as a scientist, as well as her wish to acknowledge the patient perspective. She fulfils the Vancouver authorship criteria apart from not being able to approve the final version of the manuscript. The inclusion of the patient as an author was also approved by the Ethics Council at Sahlgrenska University Hospital.

## Conflicts of interest

KL has received unrelated honoraria from Pfizer for participation in advisory boards. AF, HF, MDD, MK, ML and CV have no disclosures to declare.

## Data availability statement

All relevant data are included in the article. No additional datasets were generated or analysed during the current study.

## Ethics declarations

Ethical approval was not required for this case report according to international and local guidelines. A written-informed consent was obtained from the patient for publication of this case report and any accompanying images.

## Author contributions

KL and MK were responsible for the clinical care of the patient. HF was responsible for molecular pathology. CV provided supervision and methodological guidance. CV prepared the illustrations. MDD provided expertise on *PIK3CA*-targeted treatment in metastatic breast cancer. ML contributed to patient involvement. KL and AF drafted the manuscript. All authors reviewed and edited the manuscript. All authors approved the final manuscript except from AF who passed away a few weeks before its finalisation.
